# Effects of Slope Ecological Restoration on Runoff and Its Response to Climate Change

**DOI:** 10.3390/ijerph16204017

**Published:** 2019-10-20

**Authors:** Shan He, Tianling Qin, Fang Liu, Shanshan Liu, Biqiong Dong, Jianwei Wang, Hanjiang Nie

**Affiliations:** State Key Laboratory of Simulation and Regulation of Water Cycle in River Basin, China Institute of Water Resources and Hydropower Research, Beijing 100038, China; hsiwhr61@163.com (S.H.); 13586705638@163.com (F.L.); liushanshan198705@163.com (S.L.); bqdong92@126.com (B.D.); wangjw0603@163.com (J.W.); nhj199008@163.com (H.N.)

**Keywords:** slope ecological restoration, climate scenario, runoff, distributed hydrological model

## Abstract

Slope ecological restoration and climate change are important factors affecting the hydrological processes of the Huangshui River Basin in Qinghai province, China. How to quantitatively identify the impact of slope ecological restoration on runoff and whether slope ecological restoration can mitigate the impact of future climate change on runoff are both very important. In this paper, the Huangshui River above the center of Minhe county was taken as the research area, and the Pinus tabulaeformis and shrubs were taken as the main forest land types of slope ecological restoration. First, based on the law of forest land variation, the construction scales of slope ecological restoration in different periods were identified. The influence of slope ecological restoration on runoff was then quantitatively evaluated by using a distributed hydrological model. Second, the future climate scenarios of five general circulation models (GCMs) under three representative concentration pathways (RCPs) (i.e., RCP2.6, RCP4.5, and RCP8.5) from 2021 to 2050 were selected and modified by model integration. Combined with the slope ecological restoration scenarios, the influence of slope ecological restoration on runoff under future climate scenarios was explored. The results showed that the effect of slope ecological restoration was significant. Compared with 1980, the area of slope ecological restoration increased by 24% in 2017. Under the present climate conditions (1960–2017), different periods of slope ecological restoration have an effect on the process of runoff in the wet season (June, July, August, and September) and dry season (January, February, March, and December), which eliminates the maximum, replenishes the minimum, and reduces the variability of runoff processes in the watershed. Under the future climate scenario (2021–50), slope ecological restoration will reduce runoff. On the other hand, climate change will increase runoff, and the combination of the two effects will have a certain offsetting effect. On the whole, comparing the influence of slope ecological restoration on the runoff process with that of climate change in different seasons, due to the main influence of slope ecological restoration, the runoff decreased by about 55% in the temperate season (April, May, October, and November), and increased by about 50% in the dry season or wet season due to the main influence of future climate scenarios.

## 1. Introduction

Climate change and human activities (e.g., slope ecological restoration) are important factors that have affected the hydrological cycle of river basins in recent years [1,2], and they are also major research issues in the field of hydrology at present [2,3]. Rising temperatures caused by climate change are accelerating the processes of global and regional water cycles, thereby leading to changes in atmospheric circulation, precipitation, evaporation, soil moisture, and river runoff, as well as temporal and spatial change of water resources [4]. Moreover, due to the implementation of slope ecological restoration, the vegetation scale has increased, followed by the changes in surface roughness, which also directly or indirectly affect the above process [5,6]. In addition, because of the sensitivity of vegetation to climate change, vegetation plays a vital role in maintaining the climate stability of the terrestrial ecosystem [7], and the interaction of various factors on the watershed scale may have compounding effects, resulting in changes of the surface runoff process [5,8]. Therefore, considering future climate conditions, how slope ecological restoration will affect the temporal and spatial distribution of runoff and the variability of runoff process deserves further exploration.

Scholars have done considerable research on the impact assessments of climate change on regional runoff. The research has mainly focused on contribution rate analyses of historical meteorological data to runoff [9,10,11,12], analyses of climate change based on regional climate models (RCMs) [13], and assessments of the impact of climate change on runoff based on Global Circulation Models (GCMs) combined with a hydrological model [14,15,16,17]. Relevant studies show that the simulation ability of climate models will be significantly improved by schema integration [18,19]. The influences of slope ecological restoration on runoff are mainly studied by field experiments and historical data analysis. Field experiments usually investigate the effect of forest changes on runoff through long-term planting. Hatma investigated the effects of vegetation changes on the rainfall-runoff response in different types and periods of pine forest plantation based on their respective forestry treatments by experiments in pine forest and mixed-plant forest catchments. Chu et al. carried out an experiment on a eucalyptus plantation in South China to assess the effects of the enriched planting of native tree species on the surface water, soil erosion, and nutrient losses. However, the field experiments take considerable time and are limited by site scales [20,21,22]. The analysis of historical data is usually based on a regression analysis of long-term data, and a high accuracy is required for the data [7,23,24]. In recent years, there have also relevant studies that simulated the responses of different afforestation scenarios to runoff through the establishment of hydrological models [25], but most of these studies did not follow the law of forest land variation, and the definition of slope ecological restoration scope is still lacking.

The response mechanism of slope ecological restoration to runoff under future climate change is complex [1,26,27,28,29]. Most of the current research has been carried out by changing the landuse and coupling it with climate scenarios [26,30,31]. The vast majority of studies about the effects of slope ecological restoration and climate change on the water resources of the Yellow River have focused on the trends analyses of annual streamflow and the attribution of the changes in streamflow to climate change and slope ecological restoration [32,33,34]. Among various the applied approaches in the research, Budyko’s frameworks are the most frequently used [35,36]. However, this research usually does not follow the forest variation law of slope ecological restoration, and the exploration of climate change and slope ecological restoration’s relationship to runoff and the quantification of their impact on runoff have rarely been conducted [37].

The overall objective of this study is to investigate the impacts of slope ecological restoration and future climate change on runoff process for Huangshui River basin. This objective was achieved by performing the following four steps: (1) Based on the law of forest land variation, the scope of slope ecological restoration in different periods (1980, 1980–2000, 2000–07, and 1980–2017) was quantitatively identified; (2) a runoff simulation was performed for the period from 1965 to 2017 under different slope ecological restoration scenarios using the Water and Energy transfer Processes (WEP) distributed hydrological model, as well as an exploration of the impacts of slope ecological restoration on the runoff process; (3) a runoff simulation was done for the period from 2021 to 2050 under different slope ecological restoration scenarios, as well as three RCPs, the RCPs (RCP2.6, RCP4.5, and RCP8.5) are the three representative pathways from the Intergovernmental Panel on Climate Change (IPCC); (4) examining the impacts of slope ecological restoration on future runoff process (2021–50) under future climate scenarios. The results of this study should improve our understanding of variations in runoff under slope ecological restoration, and the potential of slope ecological restoration to mitigate future climate change. It will provide reference for better water resources management of the Yellow basin in the future. This management will be vital for building an ecological civilization and restoring key ecosystems to strengthen the quality and stability of the ecosystems in China.

## 2. Materials and Methods

### 2.1. Study Area

**Figure 1:**
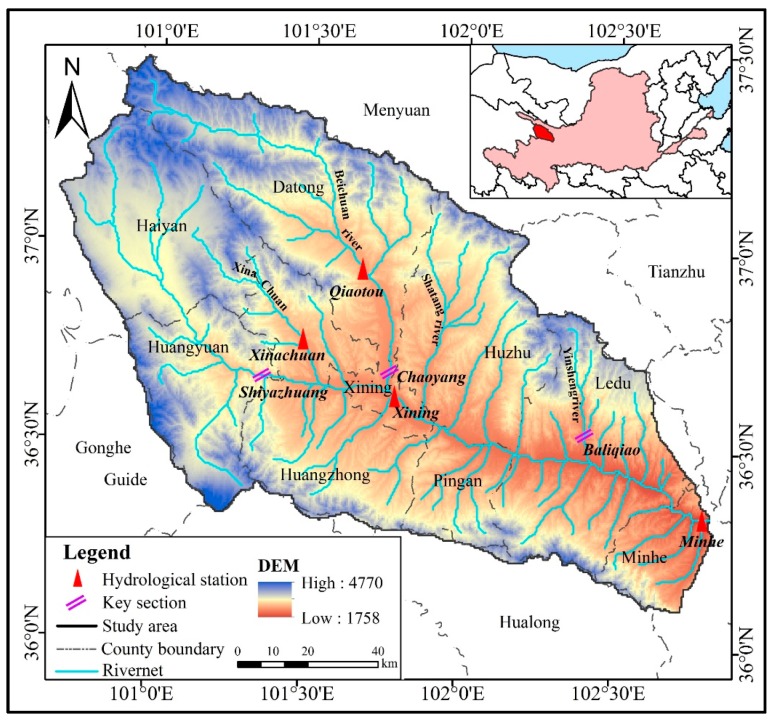
Location, elevations, hydrological stations, and key sections of the Huangshui River basin.

Huangshui is the first tributary of the upper reaches of the Yellow River in northern China, which flows through Qinghai and Gansu Provinces. The total length of the main stream is 374 km, and the area is 17733 km^2^. The study area is the Huangshui River basin above the center of Minhe County, with a total basin area of 15558 km^2^ (Figure 1). The Huangshui River Basin is located in the transition zone between the Loess Plateau and the Qinghai-Tibet Plateau, ranging from 36°02′N–37°28′N and 100°42′E–103°01′E. The basin lies in the northwest inland, far from the sea, which belongs to a plateau arid and plateau semi-arid continental climate. The annual average precipitation was 381.1 mm in the period from 1960 to 2017. The distribution is extremely uneven during the year, with the most precipitation from May to September, accounting for 84.3% of the annual precipitation. A total of 76.1% of the regional’s Normalized Vegetation Index (NDVI) reached 0.6, and the main vegetation types include coniferous forests, shrubs, grasslands and meadows, accounting for 3.6 %, 24.5%, 29.7%, and 27.9% of the whole basin, respectively. Since the 1970s, in order to effectively improve the local ecological environment and slow down soil and water loss, slope ecological restoration has been carried out through the afforestation of Pinus tabulaeformis and recovered vegetation via enclosures. By 2017, the forest land area reached 38.36%, of which 62.6% was caused by slope ecological restoration. In recent years, the regional climate has become warm and humid [38,39].

**Figure 2:**
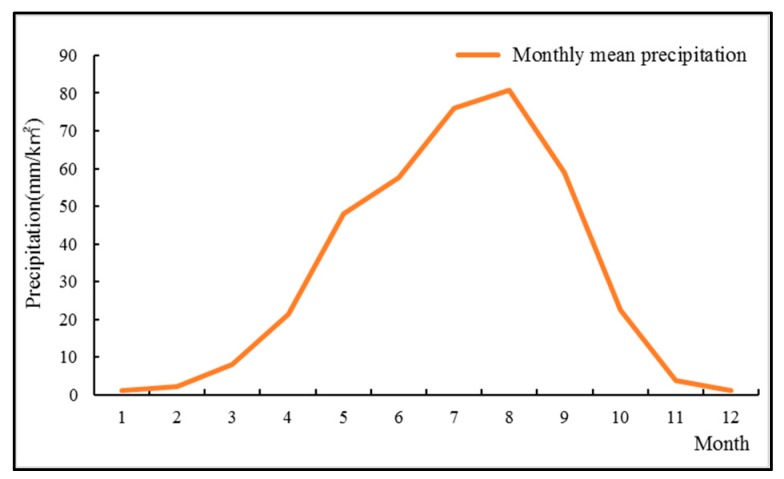
Monthly mean precipitation of the Huangshui River basin (1960–2017).

In this study, the whole year of the basin is divided into three different hydrological seasons: the wet season (June–September); the temperate season (April, May, October, November); the dry season (January, February, March, December). The seasonal definition generally occurs according to the runoff process of the river, and the runoff from small to large [40]. The division of seasons is different in different basins. Because the basin belongs to the Yellow River basin, seasonal division should refer to the division of the Yellow River’s basin season and combine with the discharge process of the study basin (Figure 2, Table 1). The result agrees with that of many researchers in the Yellow River Basin [41,42,43].

### 2.2. Data and Process

#### 2.2.1. Data Sources

The basic data required for this study mainly include meteorological data, soil data, administrative division data, landuse data, hydrological data, and future climate scenarios. (Table 2).

The representative concentration pathways (RCPS) are developed by Intergovernmental Panel on Climate Change (IPCC) for the fifth assessment report in May 2011, which provides a comprehensive set of high spatial resolution climate scenarios. In this study, three representative RCPs for future climate change conditions were used, that is, the high emission scenario RCP8.5, the medium emission scenario RCP4.5 and the low emission scenario RCP2.6 [44,45]. Furthermore, we selected data sets from five general circulation models: GFDL-ESM2M, HadGEM2-ES, IPSL-CM5A-LR, MIROC-ESM-CHEM, and NorESM1-M (Table 3). The output resolution of model is low (about 100–500 km), which limits the analysis of climate change impact directly applied to regional or watershed scale [46,47]. In this study, the data from GCMs were interpolated, bias-corrected and downscaled at a 0.5° × 0.5° spatial resolution to the WATCH Forcing Data (WFD) [48] in the fast-track of the Inter-Sectoral Impact Model Inter-comparison Project (ISI-MIP) [49]. The methods are bilinear interpolation and statistical bias-correction based on probability distribution [50,51,52]. The bias-corrected data sets could reflect changes in climate variability and the atmosphere’s mean and could better be used to investigate the effects of extreme hydrological events [53,54].

#### 2.2.2. Data Processing

**Figure 3:**
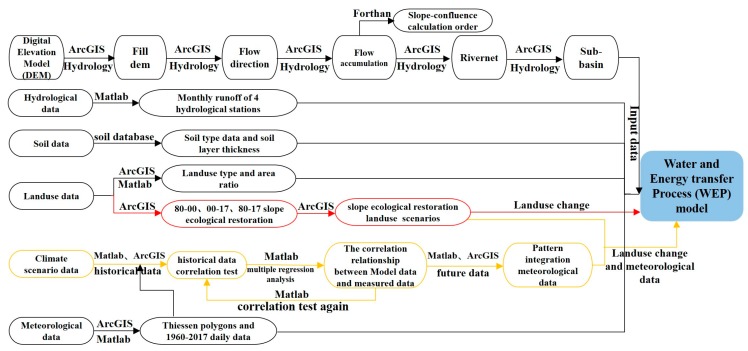
Data processing flow.

**Figure 4:**
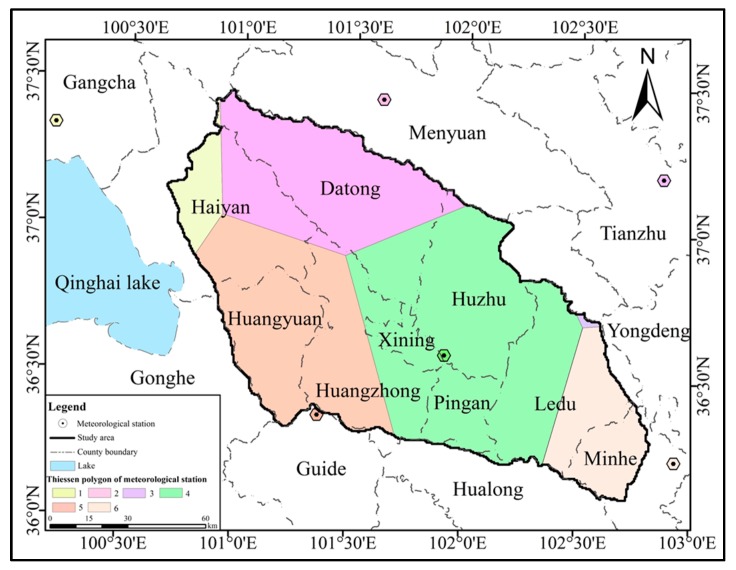
Meteorological stations and Thiessen polygons.

**Figure 5:**
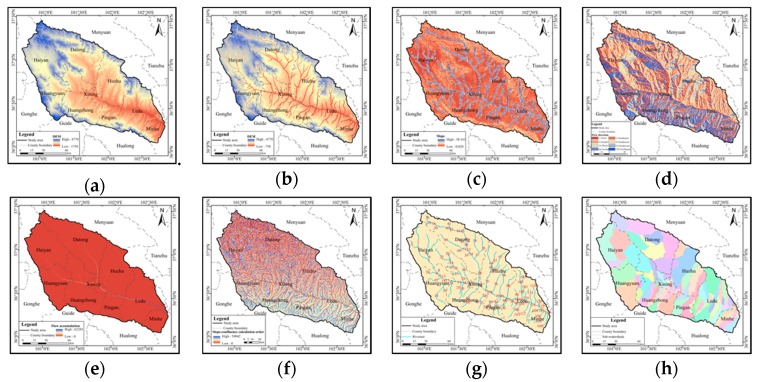
Sub-watershed and Slope-confluence calculation order formation processes ((**a**) Digital Elevation Model (DEM); (**b**) revised DEM; (**c**) slope; (**d**) flow direction; (**e**) flow accumulation; (**f**) calculation order of slope confluence; (**g**) river code; (**h**) sub-basin).

The data involved in this section were divided into three categories (Figure 3). The first category is the input data of the WEP distributed hydrological model, including the DEM, meteorological data, landuse data, soil data, which were processed by ArcGIS and MATLAB (Figure 4 and Figure 5). The second category includes the three period slope ecological restoration data for 1980–2000, 2000–17, and 1980–2017 processed by ArcGIS.

**Figure 6:**
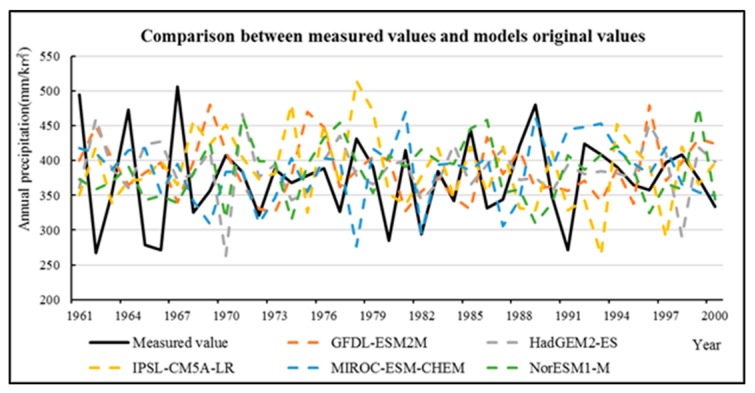
Comparison between the measured values and the five representative models original values (annual precipitation from 1961 to 2000).

**Figure 7:**
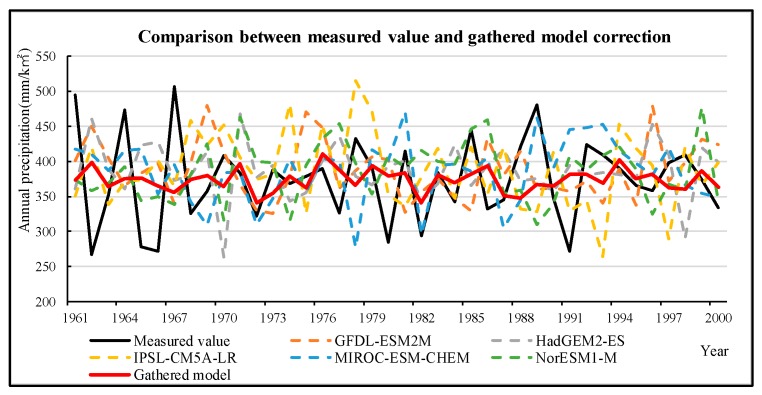
Comparison between the measured value and the gathered model correction (annual precipitation from 1961 to 2000). The red line is the gathered model value, which was closer to the measured value (black line) compared to the different model values (colored lines).

The third category includes climate scenario data. There is great uncertainty in the application of climate models: On the one hand, there are great differences between the simulation results of the model and the measured values; on the other hand, it is difficult to give the same changing trend of the simulation results of different models [55]. In addition, Shaowu et al. found that the uncertainty of the global climate model is mainly reflected in three aspects: the unpredictability of the economy, the difference of the model, and the uncertainty when using the atmospheric circulation model to drive the regional model. Among them, the difference of the model mainly manifested in that each model has its own characteristics and advantages, and the physical processes of different models are different. [56]. Therefore, in order to reduce the uncertainty of the model, the historical data for GCM are compared with the measured values for the same period (Figure 6, Table 4), and then the schema integration and data correction are performed by MATLAB based on a multiple regression analysis (Figure 7, Table 5).

### 2.3. Key Technologies and Identification Methods

This study first constructed a distributed hydrological model WEP, which simulated the runoff process in the study area from 1965 to 2017. Based on the results of the slope ecological restoration, the impacts of slope ecological restoration on the runoff in different seasons were quantitatively identified. Furthermore, we selected three RCPs including RCP2.6, RCP4.5, and RCP8.5 as representatives of high, medium and low emission scenarios. Based on the statistical analysis method, the multivariate linear regression model was established, and used to generate a set of gathered climate scenario data. Compared with the five GCMs’ data, the gathered data is more suitable for the basin. The slope ecological restoration scenarios in different periods were combined with climate scenarios to explore the mitigation effects of future climate change.

#### 2.3.1. Distributed Hydrological Model 

In this study, we use the Water and Energy transfer Process (WEP) model for the basin to simulate the hydrological cycle. The water and energy transfer processes (WEP) model [57] was developed by combining the merits of the PBSD models and SVAT models. The WEP model has the following main characteristics: (1) it combines the modeling of hydrological processes and energy transfer processes; (2) the model could directly reflect topography’s effects in runoff generation; (3) the mosaic method [58] is used in the computation unit due to the heterogeneity of landuse [52]. Previous studies have widely applied this model in different watersheds (especially in the Yellow basin) with various climate and geographic conditions [59,60,61]. Because its main characteristics are more suitable in the basin, and the model is widely used in the Yellow River Basin, the WEP model was selected for this study.

##### Model Structure and Simulation Elements

The model structure is mainly divided into a horizontal structure and a vertical structure. The vertical structure refers to the calculation of the flow generation process by simulation of the vertical structure in each basic calculation unit. The horizontal structure mainly includes channel confluence of slope runoff with tributaries or main streams. In terms of its simulation elements, the hydrological processes simulated in this study mainly include the canopy interception, and surface process, as well as the soil process, groundwater process, slope confluence and channel confluence processes. These elements all have physical mechanisms and can be expressed or calculated by the corresponding mathematical formulas.

##### Model Calibration and Validation Criteria

This study selected the Nash–Sutcliffe efficiency coefficient, the correlation coefficient, and the relative error to calibrate and verify the model. The basic criteria are as follows: (1) The average annual runoff error in the simulation period is as close as possible to 0; (2) the correlation coefficient of simulated flow and the observed flow is as close as possible to 1; (3) the Nash–Sutcliffe efficiency coefficient is as large as possible.

#### 2.3.2. Extent of Slope Ecological Restoration

**Figure 8:**
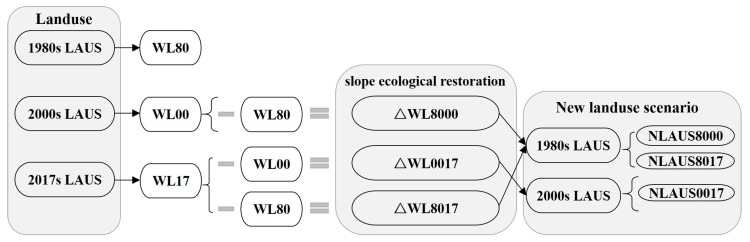
Flow chart of the slope ecological restoration scenarios constructed in different periods (1980s LAUS, 2000s LAUS and 2017s LAUS refer to landuse in 1980, 2000, and 2017, respectively; WL80, WL00 and WL17 refer to forest land in 1980, 2000, and 2017, respectively; △WL8000, △WL0017 and △WL8017 refer to slope ecological restoration in the periods of 1980–2000, 2000–17 and 1980–2017, respectively; NLAUS8000, NLAUS0017, and NLAUS8017 refer to the underlying surface scenarios considering slope ecological restoration in the periods of 1980–2000, 2000–17 and 1980–2017, respectively).

Based on the landuse in 1980, 2000 and 2017, the forest land was extracted by the tools of “Select by attributes” and “Export data” in ArcGIS. It is assumed that the forest land in 1980 (WL80) was “natural forest land”, and the newly added forest land in landuse in 2000 and 2017 was the slope ecological restoration of 1980–2000 and 1980–2017 respectively. Assuming that forest land (WL00) was “natural forest land”, the newly added forest land in landuse in 2017 was the slope ecological restoration from 2000 to 2017. By superimposing the above slope ecological restoration in the three periods in the landuse for 1980 and 2000, respectively, the underlying surface scenarios, considering slope ecological restoration in the three periods of 1980–2000, 1980–2017, and 2000–17 can be obtained (Figure 8).

#### 2.3.3. Impacts on the Historical Runoff Process

This study mainly analyzed the spatial and temporal extent effects of the slope ecological restoration on runoff in different periods. At the spatial scale, the runoff variations of seven key sections were compared to identify the spatial impacts of slope ecological restoration on the runoff. At the temporal scale, the first procedure is to analyze the annual average, wet season, temperate season, dry season and monthly average runoff from 1965 to 2017 to identify the temporal impacts of slope ecological restoration on the amount of runoff. The second procedure is to adopt a different time series (inter-annual and inter-monthly) coefficient of variation (CV) for the runoff to analyze the impacts of slope ecological restoration on the process of runoff change. CV is an indicator that effectively reflects the interannual variability of the runoff. The CV being larger indicates that the variation of the runoff is stronger [62].

#### 2.3.4. Mitigation of Future Climate Change

In this study, the three emission scenarios of RCP2.6, RCP4.5 and RCP8.5 were selected to estimate the temporal and spatial evolution trend of runoff from 2021 to 2050. Then, combining the slope ecological restoration scenarios with the climate scenarios, the influences of the three periods of slope ecological restoration on future runoff were analyzed under each climate scenario. The differences were then compared in the runoff variation under different climate scenarios, and then were explored whether slope ecological restoration had a greater mitigation effect on the extreme value process. By comparing the differences of the runoff change under different climate scenarios, we explored whether slope ecological restoration had a greater mitigation effect on the extreme value process.

## 3. Results

### 3.1. Spatial Analysis of Slope Ecological Restoration in Different Periods

**Figure 9:**
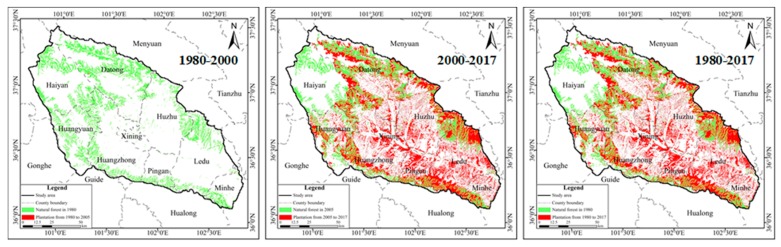
Scale of the coupled slope ecological restoration and natural forest in different periods (the red is the slope ecological restoration and the green is the natural forest).

The change trends of slope ecological restoration in different periods were as follows (Figure 9): Compared with 1980, by the year 2000, the forest land had increased relatively by 0.2%, and by 2017, the forest land had increased relatively by 131.5%; the area of the slope ecological restoration was 3735 km^2^. Thus indicated that the change in the scale of slope ecological restoration mainly happened after 2000. From an ecological point of view, the slope ecological restoration had many environmental effects, including greater carbon sequestration [63], increased soil stability and improved water quality [64].

On the whole, the scale of slope ecological restoration was generally concentrated in the downstream of the river and along the banks of the river. For the downstream distribution of Xining city and many counties, the slope ecological restoration will be beneficial to the urban ecological environment and local microclimate improvement and will also be conducive to the sustainable development of human beings and the environment.

### 3.2. Model Calibration and Validation

**Figure 10:**
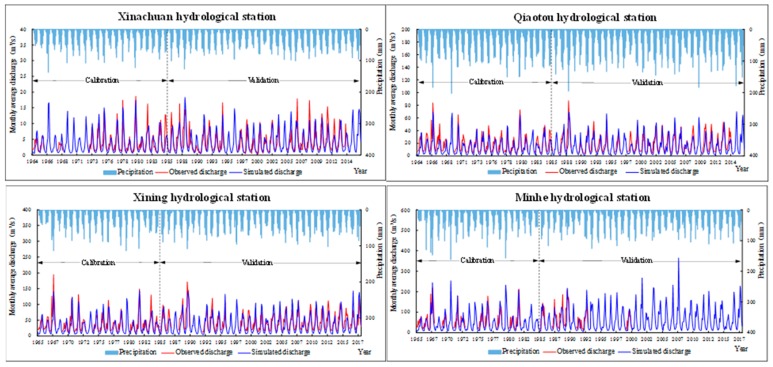
The observed and simulated monthly discharge at the four hydrological stations for the calibration period (1965–1985) and the validation period (1986–2017).

Xinachuan Station (located in the tributary of Xinachuan River), Qiaotou Station (located in the upper reaches of the tributary of Beichuan River), Xining Station (located in the middle reaches of the main stream) and Minhe Station (located in the lower reaches of the main stream) were selected to verify the monthly natural runoff process in the basin. The M-K method was used to analyze the abrupt change with a 10-year moving average precipitation in the basin, and 1985 was selected as the abrupt point, which is the demarcation point between the calibration period and the validation period. The results showed that the NSE coefficients were greater than 0.65, the correlation coefficients were greater than 0.85, and the relative errors were about 15% in the calibration period. The NSE coefficients were greater than 0.6, the correlation coefficients were about 0.8 and the relative errors were about 15% in the validation period (Figure 10, Table 6).

### 3.3. Impacts of the Different Periods of the Slope Ecological Restoration on the Runoff Process of the River Channel

**Figure 11:**
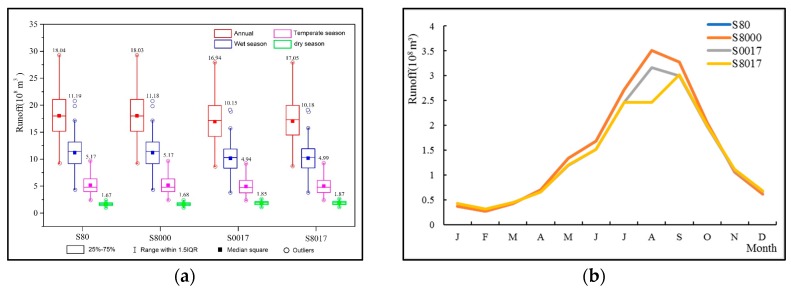
(**a**) Boxplots of the annual/seasonal runoff simulations for Minhe station under the four slope ecological restoration scenarios during 1965–2017. The number of each box represents the average. (**b**) Mean monthly runoff for Minhe station under the four slope ecological restoration scenarios during 1965–2017.

To facilitate expression, the abbreviation (S) is combined with numbers for the different periods (80, 8000, 8017) to represent the slope ecological restoration scenarios, and combined with the representative numbers of the RCPs (26, 45, 85), the slope ecological restoration scenario is represented in a certain period and a certain climate scenario (Table 7). The results of the runoff comparison from 1960 to 2017 were as follows: According to the average annual, wet season, and temperate season runoff of the whole basin, compared with the runoff simulated under S80, the runoff simulated under S8000, S0017 and S0017 decreased. The runoff of S0017 was the lowest of the three, with an average annual runoff decrease of 110 million m^3^, which was 6.1% lower than that of the S80. The smallest change in the runoff was the S8000, which was close to the runoff of the S80, with a reduction of only 1 million m^3^. The runoff in the dry season showed that, compared with the runoff simulated under S80, the runoff simulated under S8000, S0017, and S8017 increased by 0.6%, 10.78% and 11.98%, respectively. The increase of the forest land extent was conducive to an increase in runoff during the dry season (Figure 11a). Furthermore, according to the results of the monthly runoff (Figure 11b), compared with S80, the runoff of other schemes increased significantly or slightly in January, February, March, November and December, correspondingly, and the runoff of other schemes decreased from April to October. The results indicated that the slope ecological restoration had the effects of “eliminating peak and replenishing dry” during the process of runoff.

**Figure 12:**
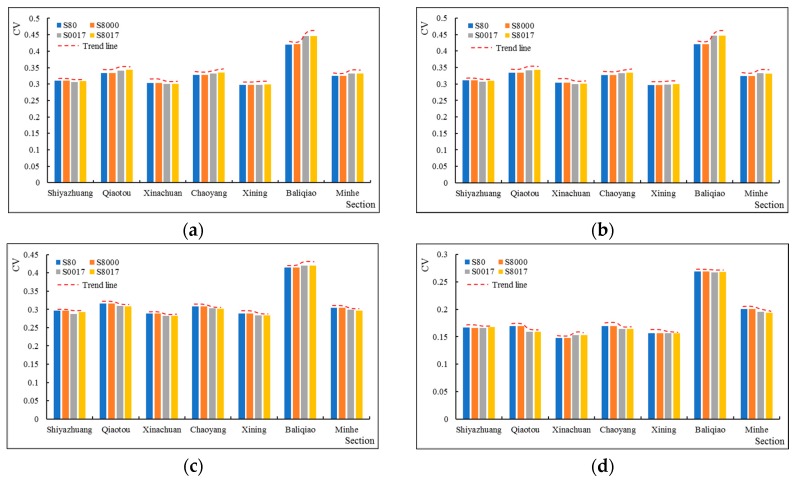
(**a**) Comparison of the annual CV for seven sections under different slope ecological restoration scenarios; (**b**) Comparison of wet season CV for seven sections under different slope ecological restoration scenarios; (**c**) Comparison of temperate season CV for seven sections under different slope ecological restoration scenarios; (**d**) Comparison of dry season CV for seven sections under different slope ecological restoration scenarios.

Furthermore, we analyzed the CV of different periods of the slope ecological restoration scenarios for the seven key sections. For the annual and wet season results, compared with the CVS80, the CVS8000, CVS8017, and CVS0017 of Shiyazhuang and Xinachuan showed a decreasing trend. The CVs in Qiaotou and Chaoyang showed a slightly increasing trend. These had effects on the downstream in Xining, resulting in no significant changes in its CV. Moreover, compared with CVS80, CVS8000, CVS8017 and CVS0017 of Baliqiao on the downstream tributary showed an increasing trend. Therefore, the CV of the Minhe near the exit of the mainstream showed a slightly increasing trend. According to the results of the temperate season, compared with CVS80, CVS8017 and CVS0017 of Baliqiao showed a slightly increasing trend, while the trend of the other sections was decreasing. Similar trends were also found for the dry season (Figure 12). Therefore, slope ecological restoration reduced the runoff variability in the temperate season and the dry season to some extent, and there was a spatial inconsistency phenomenon for the effects in the wet season.

### 3.4. Mitigation of Slope Ecological Restoration on Climate Change

**Figure 13:**
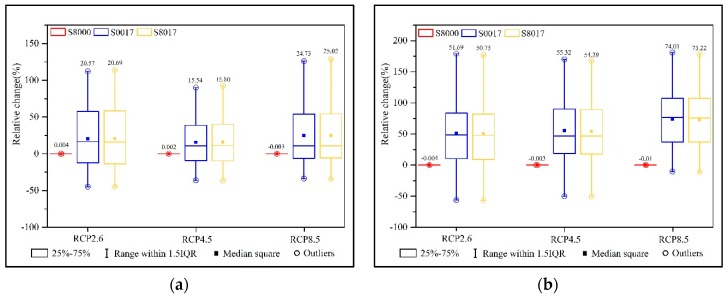

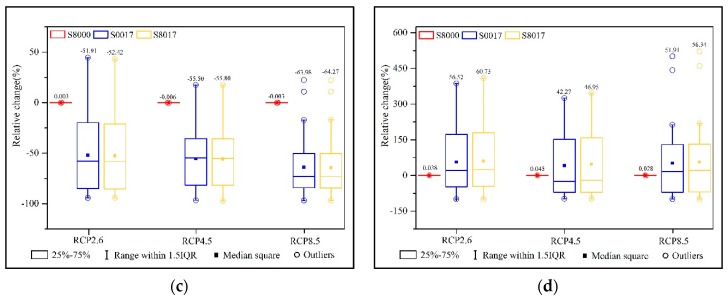
Boxplots of the relative change of (**a**) the annual runoff, and (**b**) runoff in the wet season (June, July, August, and September), and (**c**) the temperate season (April, May, October, and November) runoff, and (**d**) runoff in the dry season (January, February, March, and December) at Minhe station under three RCPs during 2021–2050. The number of each box represents the average value.

In this section, the study compared the effects of slope ecological restoration in different periods on the runoff under climate scenarios, assuming that the responsive relationship between the climate variables and runoff will remain the same [65]. By calculating the relative change in the runoff (e.g., (S8000-S80)/S80) (Figure 13), the results were as follows. Overall, under the same RCPs, the annual average runoff of the S0017 and S8017 both increased by 20%, compared with that of S80, while the slope ecological restoration reduced the average annual runoff under historical climate conditions. Thus, under the same slope ecological restoration scenarios, the runoff evolution was different due to changes in climate conditions. Therefore, the future runoff evolution in this study area was mainly affected by climate change. According to the different seasons, the runoff of S0017 and S8017 increased by more than 50% compared with that of S80 in the wet season and dry season, and reduced by about 55% in the temperate season. Therefore, under the climate scenarios, the slope ecological restoration scenarios mainly affected the runoff in the temperate season, while in the wet season and the dry season the runoff was still mainly affected by climate change.

**Figure 14:**
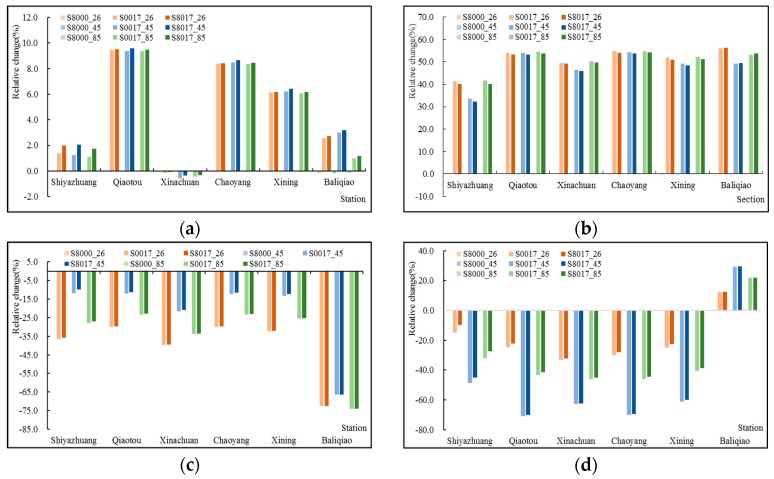
Relative change of (**a**) annual runoff, (**b**) runoff in wet season, (**c**) runoff in temperate season, and (**d**) runoff in the dry season for seven sections under three RCPs during 2021–2050.

By comparing the runoff changes of the key sections (Figure 14), under the same RCP, except for Xinachuan, the annual average runoff and the runoff in the wet season both increased compared with that of S80, while the runoff in the temperate season and dry season decreased. Under the same slope ecological restoration scenario (S8000, S8017, and S0017), the annual runoff of the three RCPs was similar, but the runoff in different hydrological seasons was different. In the wet season, the runoff of the three climate scenarios showed a trend of S45 < S26 < S85, S26 < S85 < S45 in the temperate season, and S45 < S85 < S26 in the dry season, which were basically the same as the results of S80.

**Figure 15:**
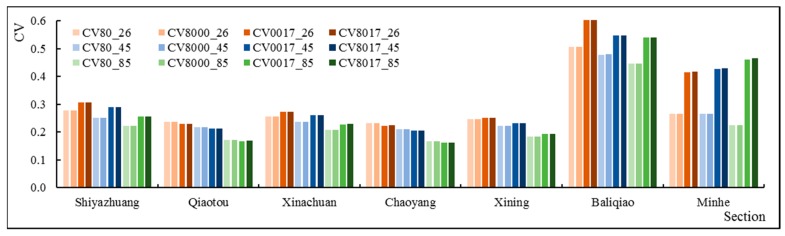
Annual runoff CV under different slope ecological restoration scenarios and different climate scenarios.

In addition, the study calculated the CV of the annual runoff (Figure 15). Under the same RCP, overall, the slope ecological restoration scenarios increased the variability of the runoff process, especially S0017 and S8017. However, the Qiaotou and Chaoyang on the Beichuan River had the opposite result, that is, the slope ecological restoration scenarios reduced the variability of the runoff process. It reduced the runoff variability of the main stream Xining Station to some extent. Under the different RCPs, no matter what kind the slope ecological restoration scenarios were, except for Minhe, the arrangement of the CVs of other sections presented a trend of CV26 > CV45 > CV85, which was the same as the results of S80 (no slope ecological restoration). According to the Minhe, the CVs of S0017 and S8017 were different from those of S80: CV85 > CV45 > CV26. The results revealed that slope ecological restoration has affected the hydrological rhythm under the future climate scenarios to some extent.

**Figure 16:**
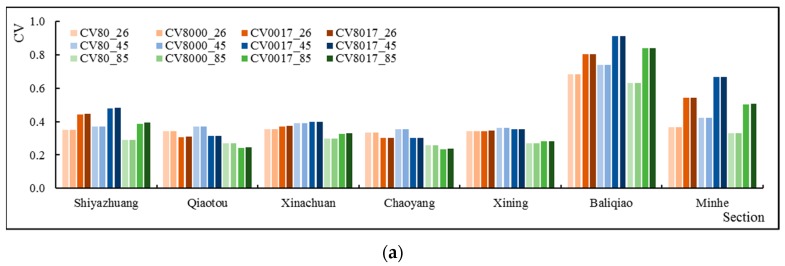

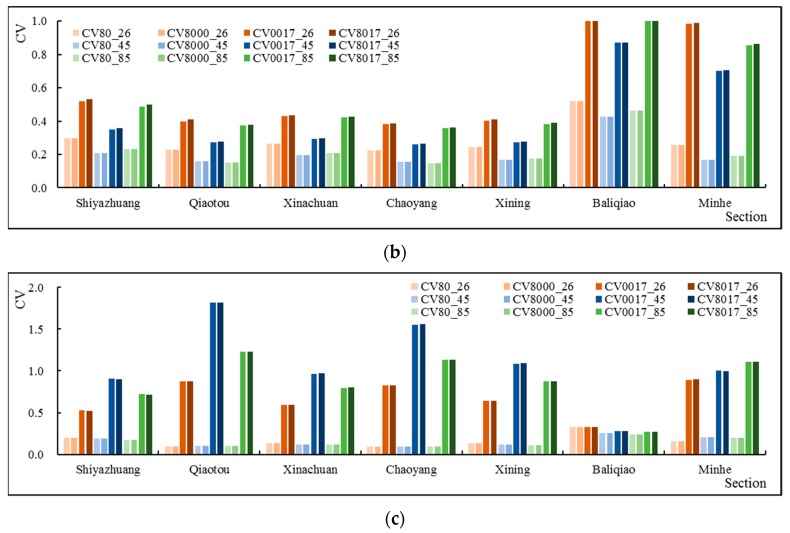
The runoff CV in the wet season (**a**), temperate season (**b**) and dry season (**c**) under different slope ecological restoration scenarios and different climate scenarios.

Furthermore, the study calculated the runoff CV in different seasons (Figure 16). The results of the wet season were similar to those for the whole year. Under the same RCP, slope ecological restoration reduced the CVs of Chaoyang and Qiaotou effectively, and also slowed the trend of increasing CVs for Xining. Under different RCPs, in the S8000, S8017 and S0017, the CVs presented in the following orders: CV45 > CV26 > CV85, which are the same as the results of S80. According to the results for the temperate season, the slope ecological restoration increased the variability of the runoff process under the same RCPs, and the results of S8000, S8017, and S0017 under different RCPs were the same as those of S80, as well. The results for the dry season were also more diverse. Under the same RCPs, the slope ecological restoration increased the CV of the runoff process of every section (except for Baliqiao). Under different RCPs, the results under S8000, S8017 and S0017 were quite different from those of S80, and the arrangement of CVs of Minhe from small to large was: CV85 > CV45 > CV26, while other sections were: CV45 > CV85 > CV26. Overall, during the next 30 years, slope ecological restoration may have a greater impact on the runoff variation process in the dry season and have less of an impact on runoff variability in the wet season and the temperate season.

## 4. Discussion

### 4.1. Analysis of the Impacts of Slope Ecological Restoration on Runoff

Because the impact of forest land changes the hydrological runoff process significantly, this study analyzed, in detail, the impact of slope ecological restoration on river runoff process in different periods. The results revealed that the slope ecological restoration in different periods was of a different scale, but, on the whole, with 2000 as the dividing point, the extent of slope ecological restoration in 1980–2000 was relatively small, and the extent of slope ecological restoration in 2000–17 was relatively large. Each slope ecological restoration scenario reduced the annual average runoff, the runoff in the wet season and temperate season. Further, the reduced runoff of S0017 and S8017 was greater than that of S8000. This is because the vegetation albedo is relatively darker, according to an increase of forest land. The extra energy could be dissipated as enhanced vegetation transpiration and soil evaporation. In addition, the slope ecological restoration can intercept precipitation, improve surface roughness, improve the soil structure, and increase infiltration, which leads to a decrease in runoff [66,67]. The results in this study are also consistent with the results of previous research in the Yellow River Basin: Sun et al. showed that plantation may reduce water yield up to 50% in the temperate zones of northern China such as the Loess Plateau [68], which is also supported by Feng et al. The study showed that the water yield reduced at a rate of 1–48 mm per year in almost 38% areas of the Loess Plateau [69]. According to the results of different seasons, for the wet season of lush vegetation, the larger the scale of slope ecological restoration, the greater the interception. For the dry season, compared with S80, the runoff of S8000, S0017, and S8017 increased. Analyzing the monthly results, the runoff of January, February, March, and December in the dry season always increased. Some studies have shown that the slope ecological restoration in the northwest of China increased the base flow [25]. In the dry season, due to the disturbance of the forest land, the soil moisture and groundwater recharge also increased, resulting in an increase in the baseflow [70]. Therefore, from the perspective of runoff, the slope ecological restoration had the effect of “eliminating peak and replenishing dry” and reduced the variability of the runoff process, which will also help reduce flood risk and drought risk, increase the amount of water available in the dry season, and contribute to the healthy development of human beings.

### 4.2. Analysis of the Spatial Impact of Slope Ecological Restoration on Runoff Variability

Compared with S80, the results of S8000, S0017, and S8017 showed an increase in the runoff variability in Shiyazhuang and Xiachuan which was located in the upper reaches above Xining, but the Qiaotou and Chaoyang showed an increasing trend. Therefore, the change of the CV in Xining was not obvious. The CV of Baliqiao located in the downstream basin below Xining Station, increased significantly, which led to a slight increase in Minhe. According to the results in the different seasons, the results of the wet season showed the same trend as the whole year, while the results in the temperate season and dry season showed a decreasing trend in the CV. Comprehensively, the increase in the annual CV of Qiaotou and Chaoyang were mainly due to the impact of the wet season, which may be due to the fact that grassland accounts for the majority of the landuse categories in the Qiaotou and Chaoyang watershed. Moreover, the forest land proportions were relatively small, so the effects in the wet season were weak. In addition, there are many factors affecting the CV, such as precipitation, ice and snow melt water. Upstream of the Beichuan River, where Qiaotou and Chaoyang are located, is in the transition zone between the Loess Plateau and the Tibetan plateau. It has hydrological characteristics of the alpine regions and snow cover. The problem, however, is more complicated. Studies have shown that after forest disturbances, greater snow accumulation is estimated (melting earlier and faster [71]), so the variability of runoff in the wet season caused by snow melting is not significantly reduced. Therefore, from the perspective of runoff variability, slope ecological restoration has the effect of “eliminating peak and replenishing dry” and presents a certain spatial heterogeneity.

### 4.3. Analysis of the Mitigation of Slope Ecological Restoration on Future Climate Change

Based on the significant results of slope ecological restoration in different periods and the effects of slope ecological restoration on the runoff process, it is important to further explore the impacts of slope ecological restoration on the runoff under future climate scenarios. Relevant studies have shown that climate change and slope ecological restoration might have the effects of counteracting or canceling, which may lead to a relative stable trend of annual runoff [26,28]; Cuo et al. showed that climate change may have a greater impact on hydrological regimes than slope ecological restoration [72]. This study has found that, under the same RCP in the future, with an increase in the slope ecological restoration scale, the annual runoff of the basin will increase by about 20%, while under the current climate conditions, the slope ecological restoration reduced the annual runoff amount by about 6.1%. Overall, the effect of slope ecological restoration is eliminated, and the runoff process is mainly affected by climate change. In addition, the study area is located in alpine and semi-arid regions, and relevant studies showed that topography also plays an important role in regulating hydrology processes. The plateau is not active in the rainfall-runoff process and is dominated by vertical hydrological processes (i.e., evaporation and percolation) [73,74]. With an increase in forest land, the evaporation and transpiration of the plateau increase under the future climate scenario, but decrease in the mountainous area [19], all of which suggests that the effect of topography on runoff processes may be greater than that of the slope ecological restoration.

According to the results in different seasons, the runoff increased in the wet and dry season, and decreased in the temperate season. Overall, the wet season led to an increase in the annual runoff, and under the climate scenarios, the precipitation mainly increased in the wet season. More precipitation may be converted to lateral flow rather than into evaporation [19]. In addition, it was found that compared with slope ecological restoration, variation in the hydrological cycle is primarily controlled by interannual precipitation [75]. All these factors led to an increase of the runoff in the wet season. Therefore, based on the increase of the runoff in the wet season, the CV is larger and the variability of the runoff increased.

## 5. Conclusions

This study explored the impact of slope ecological restoration on runoff by identifying the scale of the slope ecological restoration in different periods, and further explored the response of slope ecological restoration scenarios to climate change in combination with climate scenarios. The main conclusions are as follows:

The scale of the slope ecological restoration is significant. Compared to 1980, the area of the slope ecological restoration in 2017 was 3736 km^2^, and the area was changed primarily after 2000. In the spatial distribution of the slope ecological restoration, the patches of the slope ecological restoration are fragmented and the spatial density is inconsistent.

The slope ecological restoration in different periods has an obvious impact on the runoff process. The slope ecological restoration significantly reduced the runoff for the whole year, the wet season, and the temperate season but increased the runoff during the dry season. From the perspective of the CV value, the influence of slope ecological restoration on the runoff variation is spatially inconsistent. Overall, the slope ecological restoration effectively reduced the variability of the surface runoff process in the temperate and dry seasons.

Under the conditions of climate change, the effect of slope ecological restoration on runoff is more complicated. Under the same RCP, the slope ecological restoration mainly affected the runoff process in the temperate season, while the runoff process in the wet season and dry season are significantly affected by future climate change. Due to the counteracting or canceling effects of climate change and slope ecological restoration, the variation of the CV in different sections presented spatial inconsistency, and the CV of some sections decreased, indicating that slope ecological restoration has a certain slowing effect on future climate change.

Through quantitative identification of the scale of slope ecological restoration in different periods and simulation of the impact of slope ecological restoration on the runoff in different periods, this study preliminarily identified the response process of runoff to future climate change. These results are expected to provide insights into the feasibility of planning for future slope ecological restoration and could be useful to study the extreme effects caused by climate change in the future, with important implications for the ecological environment and the healthy development of human beings in China and globally. However, the specific impact mechanisms behind the runoff generation process and the confluence process remain unclear. Further research should be carried out in-depth, and a control experiment could be used to analyze how to affect the runoff production process and the confluence process under certain changes of precipitation, temperature and afforestation scales. In this way, the response mechanisms of slope ecological restoration to future climate change can be better explored.

## Figures and Tables

**Table 1:** Comparison of the runoff in different seasons.

Index	Wet Season	Temperate Season	Dry Season
Month	6–9	4, 5, 10, 11	1, 2, 3, 12
Range of runoff (m^3^/s)	68.15–104.34	54.15–104.89	24.67–32.52
Percentage of the year	47.48	38.43	14.09

**Table 2:** Data types, sources and description.

Data Type	Data Name	Data Source	Description
Topographic data	Digital Elevation Model (DEM)	National Geomatic Centre of China	Resolution is 90 × 90 m
Meteorology	Surface temperature	China Meteorological Data Service Center	Select daily data of the 6 meteorological stations (1960–2017)
	Relative humidity		
	Wind speed		
	Precipitation		
	Sunshine duration		
Soil	Types and Physical properties	China Soil Data Survey, China Soil Database	Reclassification according to soil category of soil database in China
Administrative division	City, county and village distribution	National Geomatic Centre of China	defined and published by the State Council of the People’s Republic of China and provincial government
Landuse	Landuse types	National Geomatic Centre of China and Department of Nature Sources of Qinghai Province, China	Landuse in 1980, 2000 and 2017
Hydrology	Location of hydrological station and reservoirs, runoff volume	Water Resources Department of Qinghai Province, China	Monthly runoff volume of the four hydrological stations (1965–2017)
Future climate scenarios	Precipitation, average temperature, average relative humidity, wind speed, solar radiation	Intergovernmental Panel on Climate Change (IPCC); Inter-Sectoral Impact Model Inter-comparison Project (ISI-MIP)	Daily data from 2021–50

**Table 3:** Information on the five general circulation models (GCMs) used in this study provided by ISI-MIP.

Modeling Center	Country	Model
Geophysical Fluid Dynamics Laboratory (GFDL)	United States	GFDL-ESM2M
Hadley Centre for Climate Prediction and Research, Met Office	England	HADGEM2-ES
L’Institut Pierre-Simon Laplace (IPSL)	France	IPSL-CM5A-LR
Technology, Atmosphere and Ocean Research Institute, and National Institute for Environmental Studies	Japan	MIROC-ESM-CHEM
Norwegian Climate Centre	Norway	NORESM1-M

**Table 4:** Index comparison between the measured values and the five representative models original values (monthly precipitation from 1961 to 2000).

Index	GFDL-ESM2M	HADGEM2-ES	IPSL-CM5A-LR	MIROC-ESM-CHEM	NORESM1-M
Correlation coefficient	0.832	0.827	0.825	0.850	0.841
Nash efficiency coefficient (NSE)	0.658	0.652	0.639	0.697	0.679
Relative error (%)	3.709	3.535	3.758	3.156	3.210

**Table 5:** Index comparison between measured value and gathered model correction (monthly precipitation from 1961 to 2000).

Index	Gathered Model
Correlation coefficient	0.888
NSE	0.788
Relative error (%)	−0.002

**Table 6:** Calibration and validation results for the monthly runoff at hydrological stations.

Station Name	Parameter	Calibration Period (before 1985)	Validation Period (after 1985)
**Xinachuan**	NSE	0.74	0.63
	R^2^	0.88	0.76
	Relative error(%)	−0.70	−7.58
**Qiaotou**	NSE	0.72	0.72
	R^2^	0.85	0.86
	Relative error(%)	−16.71	−12.94
**Xining**	NSE	0.70	0.64
	R^2^	0.84	0.83
	Relative error(%)	−12.37	−10.50
**Minhe**	NSE	0.65	0.62
	R^2^	0.86	0.84
	Relative error(%)	−12.23	−15.28

**Table 7:** Description of Abbreviations.

Abbreviation	Description
S80	No slope ecological restoration scenario
S8000	1980–2000 slope ecological restoration scenario
S0017	2000–2017 slope ecological restoration scenario
S8017	1980–2017 slope ecological restoration scenario
S26, S45, S85 S8000_26, S8000_45, S8000_85 S0017_26, S0017_45, S0017_85 S8017_26, S8017_45, S8017_85	Different climate scenarios + different periods for slope ecological restoration scenarios, e.g., S8000 represents the 1980–2000 slope ecological restoration scenario under the same climate scenario, S26 represents the RCP2.6 under the same slope ecological restoration scenarios, and S8000_26 represents the 1980–2000 slope ecological restoration scenario under RCP2.6
CV_S80_, CV_S8000_, CV_S0017_, CV_S8017_	Coefficient of variation (CV) of the slope ecological restoration scenarios in different periods
CV_2.6_, CV_4.5_, CV_8.5_	CV of the different climate scenarios
